# Prevalence and factors associated with zinc deficiency among preschool-age children in rural districts of Sidama region, Ethiopia: A community-based cross-sectional study

**DOI:** 10.1371/journal.pgph.0005356

**Published:** 2025-10-30

**Authors:** Assefa Philipos Kare, Teshome Assefa, Beruk Berhanu Desalegn, Ayalew Astatkie

**Affiliations:** 1 School of Nutrition, Food Science and Technology, Hawassa University, Hawassa, Ethiopia; 2 Nutrition, Environment and NCD Research Directorate, Ethiopian Public Health Institution, Addis Ababa, Ethiopia; 3 School of Public Health, College of Medicine and Health Sciences, Hawassa University, Hawassa, Ethiopia; University of Embu, KENYA

## Abstract

Zinc deficiency among preschool-age children is a significant global public health concern, particularly in low- and middle-income countries, with severe consequences for growth and development. However, research on zinc deficiency among preschoolers in Ethiopia, including the Sidama region, remains limited. To address this gap, we assessed the prevalence and factors associated with zinc deficiency among preschool-age children in rural Sidama. A community-based cross-sectional study was conducted from September 17 to October 3, 2024, involving 364 children aged 24–59 months. Study participants were selected via systematic random sampling. Serum zinc concentration was analyzed at the Ethiopian Public Health Institute’s nutrition laboratory, and anthropometric indices were computed using WHO Anthro 2007 Software. We collected the data using KoboCollect. Modified Poisson regression was employed to identify factors associated with zinc deficiency using Stata 17. Among the preschool-age children surveyed, 203 (55.77%) were male, with a mean age of 43.99 months (standard deviation [SD] = 12.14). The overall prevalence of zinc deficiency was 53.57% (95% confidence intervals [CI]: 48.41%–58.66%). Preschool-age children in households with five or more members had a 53% higher prevalence of zinc deficiency compared to those in smaller households (Adjusted Prevalence Ratio (APR) = 1.53, 95% CI: 1.24–1.90). The prevalence was 22% higher in children with unmet dietary diversity (APR = 1.22, 95% CI: 1.01–1.46) and 26% higher in anemic children (APR = 1.26, 95% CI: 1.04–1.51). The high prevalence of zinc deficiency among preschool-age children in rural Sidama highlights a critical public health concern. Contributing factors include larger family size, unmet dietary diversity, and anemia. Addressing this issue requires a multifaceted approach. Interventions should focus on promoting zinc-rich foods through education and diet diversification, raising awareness about birth spacing to mitigate the impact of large family size, and implementing measures to prevent and manage anemia.

## Introduction

Micronutrient deficiencies often referred to as “hidden hunger,” remain a major public health challenge in low- and middle-income countries (LMICs) [1, 2]. Among these deficiencies, zinc deficiency—recognized as a global public health concern [3–5]— is especially important due to its critical role in child growth, immune function, and overall development [6]. Zinc is an essential trace element involved in numerous biological processes, including enzymatic functions, cellular growth, and immune system regulation [7].

Inadequate zinc levels can lead to increased susceptibility to infections, impaired growth, and developmental delays [5, 8]. Zinc deficiency is associated with conditions such as growth retardation, increased morbidity from infectious diseases (especially diarrhea and pneumonia), and poor neurodevelopmental outcomes [9]. This deficiency is primarily driven by inadequate dietary intake, poor bioavailability of zinc from plant-based diets, and frequent infections that increase zinc losses [10]. The World Health Organization (WHO) recognizes zinc deficiency as a leading contributor to childhood morbidity and mortality [11].

Preschool-age children are at high risk of zinc deficiency due to their rapid growth and increased nutritional demands [12]. Globally, an estimated 17.3% of preschool children in low- and middle-income countries (LMICs) are zinc deficient, with regional prevalence as high as 23.9% in sub-Saharan Africa [5]. In Ethiopia, small-scale studies have revealed even more alarming rates, with 57.1% of infants and preschoolers affected, and the highest prevalence (67.5%) observed among children aged 54–60 months [13].

Ethiopia, like many other sub-Saharan African countries, faces a significant burden of child malnutrition, including micronutrient deficiencies [14]. This problem is most severe in rural Ethiopia, where zinc deficiency is particularly concerning due to a diet predominantly based on cereals and legumes. These staples contain high levels of phytates that inhibit zinc absorption [15]. Furthermore, limited consumption of animal-source foods, poor maternal nutrition, and inadequate complementary feeding practices exacerbate the risk of zinc deficiency among preschool-age children in these settings [16].

Several factors contribute to the high burden of zinc deficiency in Ethiopia. Socioeconomic factors such as poverty, low maternal education, and food insecurity play a crucial role in shaping dietary patterns and overall child nutrition [13, 17, 18]. Additionally, recurrent childhood infections, including diarrhea and respiratory illnesses, lead to increased zinc losses, further exacerbating deficiency rates [9]. Poor water, sanitation, and hygiene (WASH) practices also contribute to frequent infections, creating a vicious cycle of malnutrition and illness [19, 20].

Despite extensive efforts to combat malnutrition through national programs like the National Nutrition Program (NNP), zinc deficiency remains a significant public health challenge in Ethiopia. However, studies have largely focused on broader age groups, leaving preschool-age children understudied. This age group faces unique nutritional vulnerabilities due to rapid growth, developmental needs, and the transition from breastfeeding to family foods, which are often low in bioavailable zinc. Additionally, context-specific factors influencing zinc deficiency may differ for preschool-age children, underscoring the need for disaggregated data and targeted analysis. Addressing this gap is essential for guiding evidence-based interventions and informing policymakers, health professionals, and nutrition program planners. Accordingly, this study aims to estimate the burden of zinc deficiency and identify its associated factors among preschool-age children in Sidama, Ethiopia [1, 20-22].

## Methods and materials

### Ethics statement

This study adhered to the ethical principles as outlined in the Declaration of Helsinki [23], throughout the study. Ethical clearance was obtained from the Institutional Review Board (Ref No: IRB/270/16; date 18/06/2024) of the College of Medicine and Health Sciences, Hawassa University. Subsequent to the approval, an official letter of cooperation written by Sidama region Health Bureau was dispatched to relevant bodies in the data collection areas. Written informed consent was obtained from all participating mothers of preschool-aged children prior to data collection, which included questionnaires, anthropometric assessments, and venipuncture for blood samples. Mothers of preschool-age children were briefed on the study’s general purpose, potential risks, and benefits. To maintain confidentiality, participant data were linked to a unique code number. All mothers/caretakers of preschool-age children were informed of test results, and undernourished children were referred to the health post in each *kebele*.

### Study design and setting

**Fig 1:**
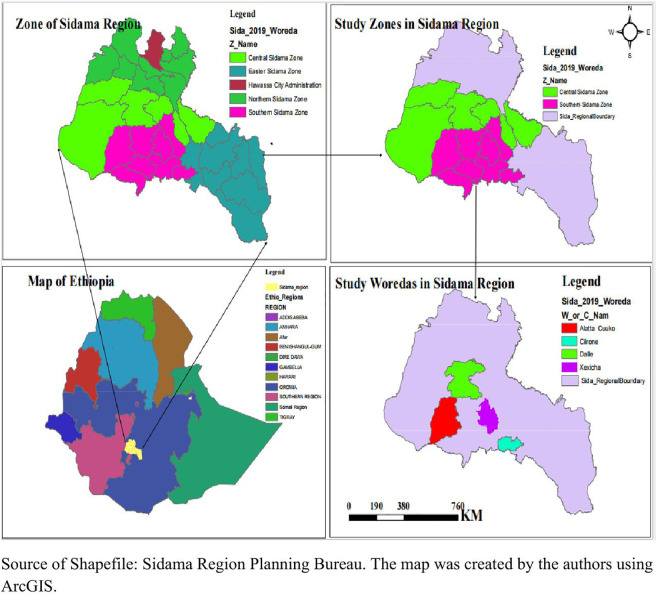
Map of the study area in the Sidama region, Ethiopia. The map shows the location of the Sidama region within Ethiopia, the study zones, and the study woredas (districts). The administrative boundary basemap was created in ArcGIS using shapefile data from the Humanitarian Data Exchange (dataset: ETH - Subnational Administrative Boundaries; https://data.humdata.org/dataset/ethiopia-cod-ab) under a Creative Commons Attribution 4.0 International License.

A community-based cross-sectional study was conducted among preschool-age children in the Sidama region of Ethiopia from September 17 to October 3, 2024. Sidama, one of Ethiopia’s twelve regional states, is situated approximately 273 kilometers south of the capital, Addis Ababa, with Hawassa City as its capital. Administratively, Sidama Region is divided into four zones and one city administration. Under the four zones there are thirty rural districts, and six town administrations (Fig 1). The region has diverse agro-ecology with highland, mid-land, and lowland weather climates. More than 85% of the population depends on a mixed crop-livestock farming system as the main means of livelihood [24]. In this system, crop production serves as the mainstay of agriculture in the region, supported by livestock rearing [25]. Farmers cultivate diverse crops including staple cereals (barley, maize, wheat, and teff), enset, and root and tuber crops, along with a variety of vegetables and fruits [26, 27]. The livestock component provides essential meat and dairy products while enhancing overall farm productivity [24]. Additionally, they grow a diverse assortment of vegetables and fruits. The diet in the region is traditionally based on kocho (a fermented starch from the enset plant) and maize, which are often processed through fermentation, a method known to reduce phytate content to some degree [15, 28, 29]. The Location map of the study area is illustrated in Fig 1.

### Population

Based on the 2007 estimation report provided by the Central Statistical Agency of Ethiopia, the total population of Sidama region is projected to be 5,301,868 individuals, with 2,953,043 (49.57%) being males and 2,636,559 being females in the year 2024 [30]. The preschool-age population in the region is estimated to be 553,038.00, comprising 274,141 males and 278,897 females [30].

This study focused on preschool children in rural communities of the Sidama region. Eligibility criteria required participants to have lived in the Sidama Region for at least six months to ensure that the study reflects long-term local exposures. Children with acute infection or skeletal deformities were excluded from the study, as these conditions could temporarily alter zinc metabolism, potentially skewing the assessment of chronic deficiency.

### Sample size determination

The sample size for estimating the prevalence of zinc deficiency was calculated using Epi Info version 7.2.6.0. The computation was based on a 95% confidence interval (Z = 1.96), an expected prevalence of 87.3% (p = 0.873) derived from prior research [17], a margin of error of 5% (d = 0.05), and a design effect of 1.5. After accounting for a 10% non-response rate, the final estimated sample size was 284.

For the assessment of factors associated with zinc deficiency, sample size was performed using parameters from previous studies [31, 32]. These calculations assumed 80% statistical power, a significance level (α) of 0.05, a design effect of 1.5, and a 10% adjustment for non-response. Two associated factors were evaluated: stunting and wealth index. For stunting, with an adjusted odds ratio (AOR) of 1.37 and an outcome prevalence of 15.3% in the unexposed group [17], the required sample size was 3,850. However, due to the substantial resource requirements for zinc analysis, this theoretically ideal sample size was deemed impractical, and feasibility was prioritized. For wealth index, which had an AOR of 4.02 and an outcome prevalence of 7.3% in the unexposed group, the necessary sample size was 287.

Since this study was conducted alongside two other objectives, a sample size of 383—originally calculated for estimating the prevalence of stunting within the same population (another objective)—was selected. This sample size satisfied the minimum requirements for all objectives while maintaining operational feasibility within logistical and budgetary limits. The selected sample size ensured that all study objectives were adequately supported without compromising scientific rigor.

### Study variables

The dependent variable for this study was zinc deficiency. Zinc deficiency was assessed by comparing the serum zinc levels against specific thresholds. For morning non-fasting samples, a threshold of below 65 µg/dL was used, while for afternoon non-fasting samples, a threshold of below 57 µg/dL was applied [33]. Children with serum zinc levels below these thresholds were classified as having zinc deficiency.

The Independent variables for this study included socio-demographic and economic factors, dietary intake and diversity, household food security, and anthropometric measurements as well as the serum zinc level measurement. The anthropometric indices were evaluated using the 2007 WHO standard reference values. A weight-for-height z-score below −2 standard deviations was classified as wasting. Underweight was defined as a weight-for-age z-score below −2, while stunting was identified by a height-for-age z-score below −2 [27, 28–30]. Anemia status in preschool-aged children was assessed based on hemoglobin levels, with a cutoff of **<**11.0 g/dL indicating anemia.

### Participant selection

A multistage sampling technique was used to select the study participants. Initially, two zones (Central Sidama and Southern Sidama zones) were randomly selected from the four zonal administrations using simple random sampling. Subsequently, from the two selected zones, four rural districts were chosen randomly from fourteen districts. Among these chosen districts, two were located in highland areas (Hula, and Teticha), one in the midland (Dale), and one in the lowland (Aleta Chuko).

From each agro-ecological zone, four *kebeles* were randomly selected using the lottery method. The selected *kebeles* were: Loya, Gidicho, Dagala Ganjure, and Godayo Guno from the highlands; Wicho, Magara, Soyama, and Semen Masinkala from the midlands; and Gambela, Gundo, Dibicha, and Rufo Chancho from the lowlands, totaling twelve *kebeles*. Among the selected districts, only Aleta Chuko contained lowland *kebeles* (eight in total), as the other districts had no lowland *kebeles*.

From the selected *kebeles*, we systematically sampled 383 preschool-aged children (3–5 years) using family records. The first study participant was selected using the lottery method, and in cases where caregivers had multiple eligible children, one child was randomly chosen using the same lottery technique.

### Data collection tools and procedure

#### Interviews.

A well-structured, pretested, and interviewer-administered questionnaire was used to collect data. The questionnaire was adopted after a comprehensive review of relevant literatures [13, 17, 34]. It consists of sections covering socio-demographic and economic factors, dietary intake and diversity [35, 36], household food security [37], morbidity-related factors, and anthropometric measurements [38] as well as the serum zinc level measurement [39]. The questionnaire was prepared in English and translated into *Sidaamu Afoo* by a native speaker with a master’s degree in language studies to ensure clarity and cultural appropriateness. Back-translation into English was performed by an independent language expert proficient in both languages to verify that the original meaning was preserved. Survey data were collected using the KoboCollect.

Data collectors and supervisors were recruited through an open application process, considering criteria like prior experience in data collection, and familiarity with the language, culture, and community norms. The data collection team consisted of four nutritionists with bachelor’s degrees in sciences and four senior laboratory technologists. Additionally, two supervisors, who were experts in public health nutrition, oversaw and supervised the entire data collection process.

Our study employed a multi-method approach to gather comprehensive data. First, trained interviewers collected socio-demographic information, economic indicators, and dietary patterns through structured interviews with mothers of preschool-aged children. Next, we conducted standardized anthropometric measurements - recording each child’s weight-for-age, height-for-age, and weight-for-height according to WHO growth monitoring protocols using calibrated equipment. Finally, we obtained venous blood samples from participants which were analyzed for serum zinc levels at the Ethiopian Public Health Institute’s laboratory, where strict quality control procedures were followed throughout the testing process to ensure accurate results.

To assess dietary diversity, the previous 24 hours recall method was applied [40]. The mothers/caregivers of the preschool-age children were asked to provide details about their children’s food and beverage intake from the previous day [35, 36]. Dietary diversity was assessed according to the Food and Agriculture Organization’s (FAO’s) recommended sixteen food groups, with each food group taken into account except when it is solely used as a condiment [35, 36]. Interviews were deliberately scheduled to avoid national holidays, celebrations, and fasting periods declared by religious leaders. This approach ensured that the data captured consumption patterns representative of regular circumstances, as festive and fasting periods often lead to atypical behaviors.

The respondents’ knowledge about a balanced diet, with a focus on zinc nutrition, was evaluated using a nine-item questionnaire. The questionnaire covered four key areas: two questions on general understanding of a balanced diet, three on the importance and impact of nutrition on health, two on nutritional recommendations for children, and two on healthy eating practices. Each question had different response options, allowing for a comprehensive assessment of participants’ awareness and understanding of balanced dietary practices.

Household food security status was determined using the validated Household Food Insecurity Access Scale (HFIAS), which assesses the occurrence of nine food access-related events during a 30-day recall period [41, 42]. Following standard HFIAS scoring protocols, households were classified dichotomously: those reporting no affirmative responses (“no”) across all nine items were classified as food secure, while those endorsing (“yes”) one or more items were categorized as food insecure, indicating compromised household-level food access [43].

### Anthropometric measurements

The weight of the preschool-age children in the sample was measured using a standard SECA digital scale, without shoes and with light clothing, to the nearest 100g on a flat surface. Two weight measurements were taken for each participant, and the average, rounded to the nearest 0.1 kg, was documented in the data collection form [44–47].

Height was measured against a vertical wall; buttocks, shoulders and back of the head touching the board using a measuring tape attached to a horizontal headboard that touches the highest point on the head. The headpiece of the measuring board was then pushed gently, crushing the hair and contacting the top of the head. Height measurements were recorded in meters, rounded to the nearest 0.1 cm, and taken barefoot or in thin socks. Two readings were recorded, and the average height was used as the height measurement [45–47].

### Biological sample

The collection and laboratory analysis of blood samples adhered to established protocols and guidelines [33, 48, 49]. Venous blood samples (5 mL) were collected from preschool-aged children by a trained senior laboratory technologist using strict aseptic techniques and standard phlebotomy procedures. Blood was drawn from the median cubital vein in the left antecubital fossa, with one portion processed for serum separation (zinc analysis) and another portion used for immediate hemoglobin concentration measurement in the field. This protocol ensured both sample integrity and participant safety throughout the collection and analysis process. After collection, blood samples were left undisturbed for 30 minutes to allow clotting. Clotted samples were then centrifuged at 3000 revolutions per minute for 10 minutes with balanced tubes to ensure stability. Serum was carefully aspirated using sterile pipettes, transferred to labeled containers, sealed, and temporarily stored at 2–8°C at the nearest health center or hospital. Within two hours of collection, samples were centrifuged, and serum was extracted within 30 minutes to minimize hemolysis and contamination. To maintain the cold chain, samples were transported in a cold box to Yirgalem Hospital Medical College, where they were stored at -36°C for up to two weeks. For analysis, serum samples were transported on dry ice to the Ethiopia Public Health Institute’s nutrition laboratory.

Throughout the process, whole blood samples were protected from light and high temperatures to ensure accurate zinc measurements. Laboratory analyses were performed within two weeks of collection by experienced personnel using validated methods, under the active supervision of principal investigators.

Hemoglobin levels were measured using the HemoCue 301 system, with blood collected in micro cuvettes. Hemoglobin values were adjusted for altitude according to WHO guidelines. Preschool-age children were classified as anemic if their hemoglobin levels were below 11.0 g/dL, consistent with WHO criteria for anemia in this age group.

### Serum zinc concentration analysis

Serum zinc concentrations were measured using Instrument MY21169003 (Software Version: 1.6.1.10384) following a standardized digestion and dilution protocol to ensure accuracy and reliability [49–51]. Serum samples were digested with nitric acid for 16 hours at 60°C [52] in a controlled oven to break down organic components and release zinc ions. Post-digestion, samples were diluted with distilled water to achieve a 5% nitric acid concentration [49], ensuring compatibility with the analytical instrument and consistent matrix effects. Calibration was performed using zinc standard solutions of known concentrations, run in ascending order to generate a calibration curve. Seronorm L1, a reference material, was used to validate accuracy and precision, with results monitored via a control chart [53]. The calibration curve demonstrated good linearity, with a correlation coefficient of 0.999, confirming the reliability of the quantification method [54].

Plasma samples were thawed to room temperature, briefly vortexed, and 100 µL was transferred into pre-labeled 15 mL conical tubes. A total of 0.25 mL of 70% HNO3 was added to digest the samples, which were then incubated at 60°C for 16 hours [52]. The acid lysate was diluted to 5% HNO3 using ultrapure water [49]. Zinc analysis was performed using the established calibration curve, achieving a 5% error margin and a 0.999 correlation coefficient [55]. Seronorm Trace Element Serum Reference Material was measured for zinc, and the obtained value served as quality control to validate the analysis. Plasma zinc content was determined using established methods, ensuring accuracy and reproducibility throughout the process [53].

### Data quality assurance

To ensure data quality, various measures were implemented. The data collection tool was designed to be simple and understandable, and it underwent pretesting and customization. Well-trained data collectors, after three days of intensive training, gathered the data under strict supervision. Daily review of completed questionnaires by supervisors and the principal investigator, along with regular evening feedback sessions, ensured quality. Anthropometric measurements were obtained using daily-calibrated precision scales. Electronic data collection was performed using KoboCollect to enhance data integrity by enabling real-time validation through range checks and skip logic to minimize data entry errors, automatically flagging missing or outlier responses for prompt correction, and supporting standardized data exports (CSV/Excel) to facilitate consistency checks and statistical validation.

### Statistical analysis

The data stored on the Kobo server was downloaded and subsequently exported to Stata version 17 for analysis. Data were described using frequency distributions, and measures of central tendency and dispersion.

We constructed the household wealth index through principal component analysis (PCA) following standardized protocols [56]. The index incorporated five asset domains: (1) durable household assets, (2) housing characteristics, (3) sanitation and water access, (4) agricultural and productive assets, and (5) additional wealth indicators (e.g., bank account ownership or membership in microfinance/savings groups, cooking fuel type, and kitchen facilities). All variables were dichotomized (1 = ownership; 0 = non-ownership) prior to analysis. Variables demonstrating substantial commonalities (>0.5) were included in the final model [57, 58]. The first principal component, accounting for 20.55% of the total variance, was extracted and used to generate the wealth index. Households were subsequently classified into tertiles (low, middle, high) based on their scores on this component.

Dietary diversity was evaluated by consolidating the standard 16 food groups into seven nutritionally meaningful categories following FAO guidelines [59]. For each preschool-aged child, a Dietary Diversity Score (DDS) was calculated by summing the number of unique food groups consumed over a 24-hour recall period, with each food group counted once regardless of consumption frequency. Children were then classified into meeting dietary diversity (met; ≥ 4 food groups) or not meeting dietary diversity (unmet; < 4 food groups) using WHO-recommended cutoffs [36].

The knowledge assessment questions had varying response formats, which were converted into binary values for analysis (correct answers = 1, incorrect/missing responses = 0). A total knowledge score was calculated by summing correct responses, with possible scores ranging from 0 to 9 (higher scores indicating greater knowledge). Respondents were categorized as having “good knowledge” if they scored ≥75% (≥6.75, rounded to 7 points) or “poor knowledge” if below this threshold. The proportions of participants in each knowledge category were then computed to assess the population’s overall understanding of balanced nutrition.

We employed the modified Poisson regression with robust error estimates to quantify associations between socioeconomic, demographic, and dietary factors (independent variables) and the binary outcome of zinc deficiency (coded 1 = present, 0 = absent), as this approach provides three key advantages over logistic regression: (1) it directly estimates interpretable prevalence ratios (PRs) rather than odds ratios (ORs), avoiding the overestimation of effect sizes when outcome prevalence exceeds 10% [60, 61] - particularly relevant given our observed outcome prevalence of 53%; (2) it delivers statistically efficient, unbiased PR estimates without the convergence challenges of log-binomial regression [62]; and (3) it yields epidemiologically meaningful measures of population-attributable risk that directly inform public health interventions [63], with cluster-robust standard errors accounting for sampling design.

Our analysis employed a staged approach to address confounding while optimizing model specification. Bivariable modified Poisson regression with robust variance estimates was employed to identify candidate variables (p < 0.25) for inclusion in the final multivariable modified Poisson model, which estimated APRs for zinc deficiency [64]. This conservative approach minimizes Type II errors in confounder selection [65].

For the multivariable analysis, we performed comprehensive multicollinearity diagnostics using variance inflation factors (VIF) prior to model fitting. Variables with a VIF > 2.5 were iteratively excluded [66, 67]—a substantially below the conventional cutoff of 10 to ensure strict independence of associated factors [68].

Final model selection employed backward elimination with retention criteria of p < 0.05 (two-tailed), supplemented by AIC-based comparison of non-nested models to ensure optimal fit [69]. We reported APR with 95% confidence intervals rather than odds ratios to avoid interpretive distortion [60]. We presented our findings in written explanations, tables with numbers, and easy-to-understand graphs

## Results

### Socio-demographic and economic information about study subjects

Of the 383 mothers of preschool-aged children approached for participation, 364 (95.0%) consented to take part in the study along with their children. Among the children, 203 (55.8%) were male. The children’s mean (± standard deviation [SD]) age was 43.99 months (±12.14), with 180 (49.5%) aged 48–59 months.

Among the mothers, 138 (37.9%) were between 25 and 29 years old, and 281 (77.2%) were housewives. For the fathers, 265 (72.8%) were farmers. In terms of education, 170 (46.8%) of the fathers and 154 (42.3%) of the mothers had no formal education.

Most participants, 343 (79.4%), belonged to the Sidama ethnic group, and 326 (75.5%) identified as Protestant. All respondents were married, with 208 (57.1%) reporting a family size of five or more members. Household-level data revealed that 161 (44.2%) experienced food insecurity, while 189 (51.9%) were classified as poor based on wealth index. Additionally, 100 (27.5%) resided in lowland agro-ecological zones. The socio-demographic characteristics of preschool-age children and parents are presented in Table 1.

**Table 1:** Socio-demographic and economic details of the study subjects in Sidama region, Ethiopia, 2024.

Variable (n = 364)	Frequency	Percentage (%)
Sex of child		
Male	203	55.77
Female	161	44.23
Age of child in months		
24–35	79	21.70
36–47	105	28.85
48–59	180	49.45
Age of mothers in years		
< 25 years	44	12.09
25–29	138	37.91
30–34	97	26.65
35 & above	85	23.35
Educational Status of father		
Not formal education	170	46.70
Formal education	194	53.30
Educational Status of mother		
No formal education	154	42.31
Formal education	210	57.69
Occupation of mother		
Housewife	281	77.20
Other works	83	22.80
Occupation of father		
Farmer	265	72.80
Other works	99	27.20
Ethnicity		
Sidama	343	94.23
Others	21	5.77
Religion		
Protestant	326	89.56
Others	38	10.44
Family size		
< 5 members	156	42.86
≥ 5 members	208	57.14
HFIAS^*^		
Insecured	161	44.23
Secured	203	55.77
Wealth Index		
Rich	99	27.20
Medium	76	20.88
Poor	189	51.92
Agro-ecological zone		
Low land	100	27.47
Mid land	129	35.44
High land	135	37.09

* HFIAS: Household Food Insecurity Access Scale

### Dietary diversity, anthropometric measurement, and health related factors of preschoolers

Among the 364 preschool-age children, 117 (32.14%) experienced unmet dietary diversity within the previous 24 hours. The median hemoglobin concentration was 12 g/dl (IQR: 2.5 g/dl) with 162 preschool-age children (44.51%) diagnosed with anemia. Examining anthropometric measurements, 191 (52.62%) were identified as stunted, 135 (37.09%) as underweight, and 82 (22.59%) as wasted. The study found 286 (78.57%) of the mothers exhibited poor knowledge of balanced and diverse dietary practices. The Dietary diversity, anthropometric measurement, and health related factors of preschoolers are presented in Table 2.

**Table 2:** Dietary diversity, anthropometric measurement, and health related characteristics of the study subjects in Sidama region, Ethiopia, 2024.

Variable (n = 364)	Frequency	Percentage (%)
Dietary diversity		
High	247	67.86
Low	117	32.14
Anemia		
Yes	162	44.51
No	202	55.49
Stunting		
Yes	191	52.62
No	172	47.38
Underweight		
Yes	135	37.09
No	229	62.91
Wasting		
Yes	82	22.59
No	281	77.41
knowledge of balanced diet		
Poor	286	78.57
Good	78	21.43

### Prevalence of zinc deficiency among preschool-age children

**Fig 2:**
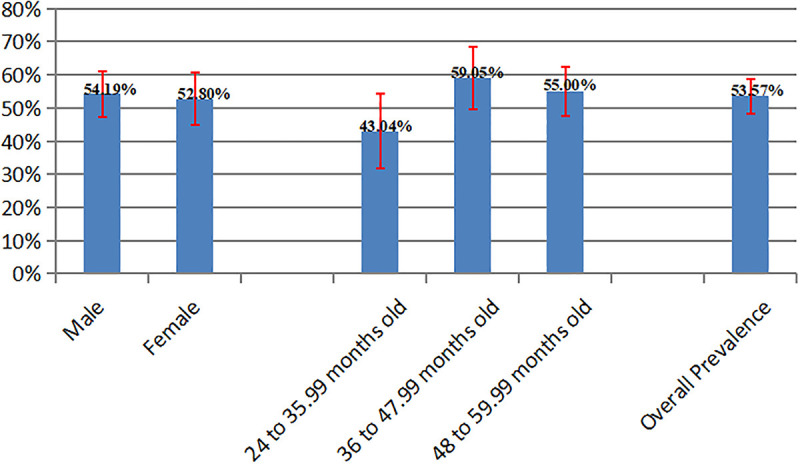
Prevalence of zinc deficiency stratified by sex and age group. Bar graph comparing the proportion of participants with zinc deficiency across different demographic subgroups (males, females, and various age categories).

The median serum zinc concentration was 61.62 µg/dL with an interquartile range (IQR) of 30.81 µg/dL. The overall prevalence of zinc deficiency was 53.6% (95% CI: 48.4%–58.7%). Prevalence was slightly higher in males than females, and highest in children aged 36–47 months. The prevalence of zinc deficiency by sex and age group is illustrated in Fig 2.

### Factors associated with zinc deficiency

Bivariable analysis of factors associated with zinc deficiency among preschool-aged children identified multiple candidate variables for multivariable analysis, including socioeconomic characteristics (wealth index, family size), maternal characteristics (age, education), child characteristics (age, anthropometric indices [HAZ, WAZ]), and nutritional characteristics (DDS, anemia status).

The modified Poisson regression confirmed larger household size, inadequate DDS, and anemia as factors associated with zinc deficiency after adjustment. Children from households with ≥5 members had a 53% higher prevalence of zinc deficiency compared to those from smaller households (APR = 1.53, 95% CI: 1.24–1.90). Similarly, those with unmet DDS (<4 food groups) had a 22% higher prevalence (APR = 1.22, 95% CI: 1.01–1.46), and anemic children exhibited a 26% higher prevalence (APR = 1.26, 95% CI: 1.04–1.51) than their non-anemic counterparts. No other factors, including wealth Index, age of mother, age of child, mother education, HAZ, and WAZ were significantly associated with zinc deficiency in the adjusted model. The factors associated with zinc deficiency among preschool-age children are presented in Table 3.

**Table 3:** A bivariate and multivariate modified Poisson regression analysis output of the factors associated with zinc deficiency among preschool-age children in Sidama region, Ethiopia, 2024.

Variable	Zinc deficiency		CPR	APR (95% CI)
	Not deficient	Deficient		
Wealth Index				
Rich	56 (33.14)	43 (22.05)	1	1
Medium	38 (22.49)	38 (19.49)	1.15 (.84, 1.58)	1.06 (.78, 1.45)
Poor	75 (44.38)	114 (58.46)	1.39 (1.08, 1.79)	1.20 (.92, 1.55)
Family Size				
< 5 members	91 (53.85	65 (33.33)		1
≥ 5 members	78 (46.15	130 (66.67)	1.5 (1.21, 1.86)	1.53 (1.24,1.90)**
Age of mother				
< 25 years	27 (15.98)	17 (8.72)		1
25–29 year	62 (36.69)	76 (38.97)	1.43 (.95, 2.13)	1.36 (.92, 2.03)
30–34 year	47 (27.81)	50 (25.64)	1.33 (.88, 2.03)	1.38 (.91, 2.09)
35 & above	33 (19.53)	52 (26.67)	1.58 (1.05, 2.39)	1.46 (.97, 2.18)
Age of child				
24–35 month	45 (26.63)	34 (17.44)		1
36–47 month	43 (25.44)	62 (31.79)	1.37 (1.02,1.85)	1.34 (1.00, 1.80)
48–59 month	81 (47.93)	99 (50.77)	1.28 (.96, 1.70)	1.21 (.91, 1.62)
Mother education				
Not formal	66 (39.05)	88 (45.13)	1.12 (.93, 1.36)	1.17 (.97, 1.41)
Formal	103 (60.95)	107 (54.87)	1	1
DDS				
Met	46 (72.78)	71 (63.59)	1	1
Not met	123 (27.22)	124 (36.41)	1.21 (.998, 1.46)	1.22 (1.01, 1.46)*
Anemia				
No	107 (63.31)	95 (48.72)	1	1
Yes	62 (36.69)	100 (51.28)	1.31 (1.09, 1.59)	1.26 (1.04, 1.51)*
HAZ				
Normal	125 (73.96)	155 (79.90)	1	
Wasted	44 (26.04)	39 (20.10)	.86 (.67, 1.10)	1.06 (.97, 1.17)
WAZ				
Normal	149 (88.17)	178 (91.28)	1	
Stunted	20 (11.83)	17 (8.72)		1.01 (.89, 1.14)

APR: Adjusted Prevalence Ratio; CI: Confidence Interval; CPR: Crude Prevalence Ratio; DDS: Dietary Diversity Score; HAZ: Height for age Z-score; WAZ: Weight for Age Z-score; *Significant association (p < 0.05); **Highly significant association (p < 0.01).

## Discussion

The overall prevalence of zinc deficiency was 53.57% (95% CI: 48.41% - 58.66%). The multivariable modified Poisson regression analysis outputs showed that family size, dietary diversity, and anemia were factors associated with zinc deficiency.

The prevalence of zinc deficiency was found to be 53.57%, indicating a severe public health burden among preschool-age children in the study area. This finding aligns with the results of a previous study [13] which reported a similar prevalence of 57.1%. The median serum zinc concentration of 61.62 µg/dL (IQR = 30.81) falls below the threshold for adequacy, underscoring the severity of the issue [33]. This level of deficiency has dire consequences. Zinc is fundamental for immune competence and linear growth; a prevalence exceeding 50% suggests a high population-level risk for increased morbidity from infectious diseases like diarrhea and pneumonia, as well as a high likelihood of stunting. The resulting vicious cycle of infection and malnutrition places a significant burden on both individual child development and the local healthcare system.

The prevalence of zinc deficiency among preschool-aged children in Sidama region (53.57%) found by the present study is much higher than the national estimate of 28% [70, 71]. This disparity likely stems from the region’s unique socioeconomic and dietary conditions. First, the region’s traditional dependence on *kocho* (*enset*-based diets) [27, 72] and maize, both high in phytate (which reduces zinc bioavailability by 45–55%) [7, 10, 73, 74], contrasts sharply with more diversified urban diets that include more zinc-rich animal-source foods [75]. Second, Sidama’s predominantly rural population faces higher poverty rates [76], which limit access to animal products [77]. Third, methodological differences in zinc assessment may explain variations across studies. While our study employed serum zinc concentrations (a direct biomarker), prior research has predominantly relied on indirect methods including dietary recall, FFQs, and predictive modeling - approaches potentially limited by recall bias and food composition database inaccuracies.

Preschool-age children residing in households with a family size of five or more had a 53% higher prevalence of zinc deficiency compared to those in smaller households (less than five members). This finding aligns with previous results indicating that larger family sizes often correlate with limited access to nutrient-rich foods due to resource constraints and food insecurity [13, 17, 78-80]. Supporting this observation, a study conducted in East Gojjam reported that an increase of family size by one member was associated with a decrease of 0.83 µg/dL in serum zinc levels, further underscoring the impact of household size on children’s nutritional status [13]. The association may stem from increased competition for limited food resources, potentially leading to inadequate dietary zinc intake. This challenge is worsened in resource-poor settings with unmet dietary diversity, highlighting the need for targeted nutritional interventions [81].

The present study also found that children consuming fewer than four food groups out of seven, classified as having unmet dietary diversity scores (DDS), exhibited a 22% higher prevalence of zinc deficiency compared to those with more diverse diets. This finding highlights the critical role of dietary diversity in mitigating zinc deficiency. Unmet DDS is often indicative of limited access to zinc-rich foods, such as meat, dairy, legumes, and nuts, which are essential for maintaining adequate zinc status [82, 83]. Minimum dietary diversity has been shown to be positively associated with the mean micronutrient adequacy of the diet [83]. These results are consistent with existing literature demonstrating that dietary diversity significantly improves micronutrient adequacy, particularly in populations reliant on staple-based diets [84, 85].

Anemia was independently associated with zinc deficiency, with anemic preschool-age children exhibiting 26% higher prevalence of zinc deficiency compared to their non-anemic counterparts. This association is consistent with the well-documented interplay between zinc and iron metabolism, as both micronutrients are essential for immune function and hematopoiesis. This relationship is biologically plausible, as zinc plays a crucial role in hemoglobin synthesis and overall immune function. Zinc deficiency can impair iron metabolism, potentially leading to anemia. Conversely, anemia may reflect broader nutritional deficiencies, including inadequate zinc intake [86, 87]. The co-occurrence of zinc deficiency and anemia may exacerbate the risk of impaired growth, cognitive development, and susceptibility to infections, underscoring the need for integrated interventions that address multiple micronutrient deficiencies simultaneously [88]. However, the cross-sectional nature of this study limits our ability to establish causality—while anemia may contribute to zinc deficiency, it could also result from pre-existing zinc deficiency.

While this study identified factors associated with zinc deficiency, other factors such as age of mother, height-for-age z-score (HAZ), and weight-for-age z-score (WAZ) were not significantly associated in the multivariate analysis. Similarly, the East Gojjam study found no association between dietary diversity, anthropometric indices, and serum zinc levels. However, this contrasts with studies linking these factors to zinc status. The lack of association may reflect dietary homogeneity, reliance on low-bioavailability staple foods, or unmeasured confounders like infections or genetics [89–91]. For instance, if most participants consumed similar zinc-poor diets, dietary diversity might not have varied enough to detect an effect. Additionally, frequent infections—common in this setting—could mask the relationship between anthropometry and zinc status by altering zinc metabolism and absorption. Genetic variations in zinc transporters or inflammatory responses may also play a role but were not assessed in this study.

The present study has some limitations. The cross-sectional design prevents determination of causality or the sequence of events. Although we found a significant association between anemia and zinc deficiency, we cannot determine which condition precedes the other. Longitudinal studies are needed to clarify the temporal relationship between these two conditions. Additionally, there is a possibility of selection bias due to the exclusion of children with acute infection or skeletal deformities, and unmeasured confounding factors such as genetic differences or undiagnosed infections may have influenced the observed associations.

## Conclusion

The high prevalence of zinc deficiency among preschool-age children in rural Sidama underscores a pressing public health issue. Larger household size, unmet dietary diversity, and anemia were significantly associated with this deficiency. Addressing the high prevalence of zinc deficiency among preschool-aged children in rural Sidama requires a multifaceted approach. This includes promoting zinc-rich foods (such as meat/oysters/ and legumes, cooked chickpeas, cooked lentils) through community education and diet diversification, raising awareness about the importance of birth spacing and limiting number of children by highlighting the nutritional challenges linked to large family sizes, and implementing interventions to prevent and manage anemia. Tackling these interrelated factors will be essential for improving child nutrition and overall health outcomes in the region.

## Supporting information

S1 Text Ethical approval letter from the Institutional Review Board (IRB). Official ethical clearance document (Ref No: IRB/270/16; dated 18/06/2024) issued by the Institutional Review Board of the College of Medicine and Health Sciences, Hawassa University.
(PDF)

S1 Data Analyzed dataset underlying the study findings. The Stata data file contains the anonymized individual-level data used for all statistical analyses and to generate the figures and results reported in this manuscript.
(DTA)
