# Supplementary material for: Prevalence and factors associated with zinc deficiency among preschool-age children in rural districts of Sidama region, Ethiopia: A community-based cross-sectional study
Source: PLOS Glob Public Health. 2025 Oct 30;5(10):e0005356. doi: 10.1371/journal.pgph.0005356 (PMC12574922; doi:10.1371/journal.pgph.0005356)
Supplement: S1 Text — Official ethical clearance document (Ref No: IRB/270/16; dated 18/06/2024) issued by the Institutional Review Board of the College of Medicine and Health Sciences, Hawassa University. (PDF) [file pgph.0005356.s001.pdf]

ሀዋሳ ዩኒቨርሲቲ
ሀክምናና ጤና ሳይንስ ኮሌጅ
የምርምር ስነ-ምግባር ገምጋሚ ቦርድ

HAWASSA UNIVERSITY
COLLEGE OF MEDICINE AND HEALTH SCIENCES
Institutional Review Board

Ref. No: IRB/270/16
Date: 18/06/2024

Name of Researcher(s): **Assefa Philipos, Ayalew Astatkie (PhD, Assoc. prof.), Beruk Berhanu (PhD, Asst. prof.)**

Topic of Proposal: ***Determinants of zinc deficiency, associated factors of the deficiency with anthropometric indices and effects of nutrition education on serum zinc level among pre-school age children in rural districts of Sidama region, Ethiopia***

Dear Researcher (s)
The Institutional Review Board (IRB) at the College of Medicine and Health Sciences of Hawassa University has reviewed the aforementioned research protocol with special emphasis on the following points:

1. Are all principles considered?				
1.1. Respect for persons:	Yes	☑	No	☐
1.2. Beneficence:	Yes	☑	No	☐
1.3. Justice:	Yes	☑	No	☐
2. Are the objectives of the study ethically achievable?	Yes	☑	No	☐
3. Are the proposed research methods ethically sound?	Yes	☑	No	☐

Based on the aforementioned ethical assessment, the IRB has:

A. Approved the proposal for implementation
B. Conditionally Approved
C. Not Approved

☑ Approval period **-18 June 2024 to 17 June 2025**
☐ Element Approved: **Protocol Version No.** 1
☐ Follow up report expected in 6 **months**

Obligation of the PI:

1. Should comply with the standard international and national scientific and ethical guidelines
2. All amendment and changes made in protocol and consent form needs IRB approval
3. The PI should report SAE within 3 days of the event
4. End of the study, including the manuscript should be reported to the IRB

Yours faithfully,

Embialle Mengistie Beyene
(PhD, Asso.Professor)
Institutional Review Board Chairperson

Hawassa University College of Medicine & Health Sciences Institutional Review Board

☎: + 046 8209290 Website:
Fax: + 046 2208755 ✉ 1560 CMHS, Hawassa-Ethiopia

Scanned with ACE Scanner
